# The aquatic annelid fauna of the San Marcos River headsprings, Hays County, Texas

**DOI:** 10.3897/zookeys.618.8560

**Published:** 2016-09-19

**Authors:** McLean L. D. Worsham, Randy Gibson, David G. Huffman

**Affiliations:** 1 Freeman Aquatic Biology Station, Department of Biology, Texas State University, San Marcos, TX 78666, U.S.A.; 2 U. S. Fish & Wildlife Service, San Marcos Aquatic Resources Center, San Marcos, TX 78666, U.S.A.

**Keywords:** Annelida, Clitellata, Hirudinida, Aphanoneura, Branchiobdellida, oligochaetous clitellates, freshwater Nemertea, spring fauna

## Abstract

The San Marcos River in Central Texas has been well studied and has been demonstrated to be remarkably specious. Prior to the present study, research on free-living invertebrates in the San Marcos River only dealt with hard bodied taxa with the exception of the report of one gastrotrich, and one subterranean platyhelminth that only incidentally occurs in the head spring outflows. The remainder of the soft-bodied metazoan fauna that inhabit the San Marcos River had never been studied. Our study surveyed the annelid fauna and some other soft-bodied invertebrates of the San Marcos River headsprings. At least four species of Hirudinida, two species of Aphanoneura, one species of Branchiobdellida, and 11 (possibly 13) species of oligochaetous clitellates were collected. Other vermiform taxa collected included at least three species of Turbellaria and one species of Nemertea. We provide the results of the first survey of the aquatic annelid fauna of the San Marcos Springs, along with a dichotomous key to these annelids that includes photos of some representative specimens, and line drawings to elucidate potentially confusing diagnostic structures.

## Introduction

The San Marcos River in Hays County, Texas (29°53.505'N; 97°55.973'W) is a spring fed river supplied with physicochemically stable water from the Edwards Aquifer (Crow 2012; Musgrove and Crow 2012). The spring outflows were impounded by a low head dam in 1849 to form a small reservoir known of as Spring Lake. Spring Lake and the upper 2 or 3 km of the spring run supports a rich biotic community (Edwards and Arnold 1961, Bowles et al. 2007, Gibson et al. 2008). At present there are four species that are federally protected, threatened, or endangered, with some other endemics probably worthy of such designation; three of which are vertebrates. The Comal Springs riffle beetle (*Heterelmis comalensis* Bosse, Tuff, and Brown) is the only protected invertebrate species that occurs in the San Marcos River (SMR). Circumstances associated with the biogeographic history of the headsprings no doubt contributed to the evolution of unique and endemic species. Indeed, many of the endemic invertebrates of Spring Lake and the San Marcos Springs are generally considered marine relicts (Holsinger and Longley 1980, Hershler and Longley 1986, Gibson et al. 2008). Therefore, endemism is high for some of the invertebrate taxa; especially taxa that are poor dispersers and have long inhabited the SMR.

The first studies of invertebrates from the SMR and nearby springs issuing from the Edwards Aquifer led to the description of several new stygobionts (Benedict 1896, Ulrich 1902, Holsinger 1966, Bowman and Longley 1976, Holsinger and Longley 1980, Hershler and Longley 1986) with no attention paid to epigean invertebrate species. The first study on epigean invertebrates only reported on trichopterans. Not surprisingly, this study led to the description of a new species, *Protoptila arca* (Edwards and Arnold 1961), which was determined to be a San Marcos endemic (Edwards and Arnold 1961). Thirty additional species of trichopterans were later reported from the San Marcos (Bowles et al. 2007). More recent surveys reported additional records of species from the SMR and associated springs (Gibson et al. 2008, Diaz and Alexander 2010, Hutchins et al. 2013).

A study of the diet of the fountain darter, *Etheostoma fonticola* Jordan and Gilbert from the SMR was the first study to report on epigean invertebrates other than trichopterans, but this diet study only reported on hard-bodied invertebrates (e.g. mollusks and arthropods); additionally, recovered specimens were only identified to order (Schenck and Whiteside 1977). Despite its low taxonomic resolution, findings from this study suggested a remarkable amount of diversity, with twelve separate orders reported from the gut contents of this one species of fish. This diversity was verified by a subsequent diet study of the San Marcos salamander, *Eurycea nana* Bishop whereby numerous taxa (also largely hard-bodied forms), were reported from the SMR for the first time (Diaz 2010).

At the time of this writing, the only reports of free-living soft-bodied invertebrates from the SMR were the mention of a stygobiotic platyhelminth and a stygobiotic hirudinean (Hershler and Longley 1986, Bowles and Arsuffi 1993) and the documentation of the first gastrotrich of the genus *Redudasys* (Gastrotricha: Macrodasyida) in the Northern Hemisphere (Kånneby and Wicksten 2014). Presented herein is the first report of identified annelids from the San Marcos River, with notes on other free-living vermiform fauna; including a new distribution record for a nemertean. This report adds several species to the ever-growing list of invertebrate taxa reported from the San Marcos Springs (SMS) and SMR. Several of these appear to be undescribed taxa that likely have a restricted distribution to the physicochemically stable spring run.

## Materials and methods

Invertebrates were collected from January 2013 to August 2014. Several sampling methods were utilized, including a Ponar grab sampler, installation of nets over spring outflows, baited traps, dip netting of vegetation and substrate, and SCUBA diving with suction devices. All collected organisms were transported live to the Freeman Aquatic Biology Station at Texas State University-San Marcos. Specimens were examined under a dissecting and/or compound light microscope and were identified to lowest possible taxon using the most recent literature (Brinkhurst 1964, Brinkhurst and Jamieson 1971, Harman 1973, Spencer 1978, Hiltunen and Klemm 1980, Kathman and Brinkhurst 1998, Pinder 2010, Wetzel et al. 2015).

## Results

At least 4 species of epigean Hirudinida, 2 species of Aphanoneura, 1 species of Branchiobdellida, and 11 (possibly 13) species of oligochaetous clitellates are present in the SMR and identified herein. At least 3 species of free-living Platyhelminthes and 1 species of Nemertea were also collected. The species of Nemertea is the first record of the phylum from the SMR, though this phylum has been documented elsewhere in the Guadalupe drainage basin (Ourso and Hornig 2000). See Table 1 for list of vermiform taxa identified in this study.

**Table 1.** List of annelid and other vermiform taxa collected from San Marcos River headsprings.

Phylum	Class	Subclass	Order	Family	Genus/species	Describer
Annelida	Clitellata	Hirudinida	Arhynchobdellida	Erpobdellidae ^[†]^		
Annelida	Clitellata	Hirudinida	Rhynchobdellida	Piscicolidae		
Annelida	Clitellata	Hirudinida	Rhynchobdellida	Glossiphoniidae	*Placobdella parasitica*	Say, 1924
Annelida	Clitellata	Hirudinida	Rhynchobdellida	Glossiphoniidae	Helobdella cf. papillata	Moore, 1952
Annelida	Clitellata	Oligochaeta	Lumbriculida	Lumbriculidae	Lumbriculidae sp_1_	
Annelida	Clitellata	Oligochaeta	Lumbriculida	Lumbriculidae	Lumbriculidae sp_2_	
Annelida	Clitellata	Oligochaeta	Haplotaxida	Naididae	*Stylaria lacustris*	Linnaeus, 1767
Annelida	Clitellata	Oligochaeta	Haplotaxida	Naididae	Chaetogaster cf. limnaei	K. von Baer, 1827
Annelida	Clitellata	Oligochaeta	Haplotaxida	Naididae	Chaetogaster cf. diaphanus	Gruithuisen, 1828
Annelida	Clitellata	Oligochaeta	Haplotaxida	Naididae	Chaetogaster cf. crystallinus	Vejdovský, 1883
Annelida	Clitellata	Oligochaeta	Haplotaxida	Naididae	*Pristina leidyi*	F. Smith, 1896
Annelida	Clitellata	Oligochaeta	Haplotaxida	Naididae	*Nais pseudobtusa*	Piguet, 1906
Annelida	Clitellata	Oligochaeta	Haplotaxida	Naididae	Dero (Dero) cf. obtusa	d'Udekem, 1855
Annelida	Clitellata	Oligochaeta	Haplotaxida	Naididae	Dero (Aulophorus) cf. furcatus	Müller, 1773
Annelida	Clitellata	Oligochaeta	Haplotaxida	Haplotaxidae	Haplotaxis cf. gordioides	Hartmann, 1821
Annelida		Aphanoneura		Aeolosomatidae	Aeolosoma cf. variegatum	Vejdovský, 1884
Annelida		Aphanoneura		Aeolosomatidae	Aeolosoma cf. quarternarium	Ehrenberg
Platyhelminthes	Turbellaria		Tricladida	Dugesiidae	*Schmidtea* sp.	
Platyhelminthes	Turbellaria		Tricladida	Dugesiidae	*Dugesia* sp.	
Platyhelminthes	Turbellaria		Macrostomida			
Platyhelminthes	Rhabditophora		Seriata	Kenkiidae	*Sphalloplana mohri* ^[‡]^	Hyman, 1939
Nemertea	Enopla		Hoplonemertea	Tetrastemmatidae	Prostoma cf. graecense	Böhmig, 1892

[†] Both a stygobiotic and epigean species were collected.

[‡] This species was not collected by the authors but was included for completeness.

### Dichotomous key to Annelida of San Marcos Springs

**Table:** 

1a	Parasitic or commensal	**2**
1b	Free-living	**5**
2a (1a)	Chaetae absent	**3**
2b	Chaetae present; commensal on gastropods (in mantle cavity); body usually quite small, <4 mm	**Chaetogaster cf. limnaei**
3a (2a)	Parasitic on exterior of vertebrates	**4**
3b	Parasitic on exterior of crayfish of Family Cambaridae (Figure 1)	Order Branchiobdellida (Family **Cambarincolidae**)
4a (3a)	Parasitic on fishes; anterior sucker about half the diameter of caudal sucker; body small (<2.5 cm)	***Family*Piscicolidae**
4b	Usually parasitic on turtles; body large, (>2.5 cm)	***Placobdella parasitica***
5a (1b)	Chaetae absent	**6**
5b	Chaetae present	**7**
6a (5a)	Multiple pairs of eyes (may be discrete and not visible)	**Family Erpobdellidae**
6b	Single pair of closely spaced conspicuous eyes	***Helobdella* sp.**
7a (5b)	Dorsal chaetae absent (at least on 10 or more anterior segments)	**8**
7b	Dorsal chaetae present (Figure 2)	**10**
8a (7a)	Ventral chaetae bifid, and at least three and up to nine per bundle (Figure 3)	**9**
8b	Ventral chaetae 1 per bundle with simple point and tip curved towards posterior of worm (Figure 4); worm elongate, up to 10 cm or more in length, but usually 4-5 cm	**Haplotaxis cf. gordioides**
9a (8a)	Prostomium more conspicuous than other *Chaetogaster* spp.; only ventral chaetae present; worm usually small, total length <4 mm (Figure 5)	**Chaetogaster cf. diaphanus**
9b	Prostomium inconspicuous with cleft (Figure 6); numerous chaetae per posterior ventral bundles; worm usually relatively large, with total length ≥ 4 mm	**Chaetogaster cf. crystallinus**
10a (7b)	Dorsal chaetae usually more than 1 per bundle and found on anterior portions of worm	**11**
10b	Dorsal chaetae short, only 1 per bundle, only found on posterior of worm; ventral chaetae 1 per bundle with simple point curved posteriad; worm elongate, up to 10 cm long, usually 4–5 cm	**Haplotaxis cf. gordioides**
11a (10a)	Ventral chaetae two per bundle and with simple point (Figure 7); worm usually quite large, total length >3 cm	**12**
11b	Ventral chaetae bifid, more than two per bundle with usually 3-9 per bundle in most species (Figure 8)	**13**
12a (11a)	Prostomium modified into elongated proboscis (Figure 9)	**Lumbriculidae sp_1_** (may be two species)
12b	Prostomium inconspicuous and without proboscis (Figure 10)	**Lumbriculidae sp_2_** (may be two species)
13a (11b)	Gills present on posterior end (digitiform projections; in some cases inconspicuous)	**14**
13b	Posterior end without gills	**15**
14a (13a)	Gill fossa with two long parallel accessory palps (Figure 11)	**Dero (Aulophorus) furcatus**
14b	Gill fossa not prolonged, often continuous with gills (Figure 12)	**Dero (Dero) obtusa**
15a (13b)	Eyes present	**16**
15b	Eyes absent	**17**
16a (15a)	Prostomium with elongate proboscis (Figure 13)	***Stylaria lacustris***
16b	Prostomium protruding conspicuously over mouth, but without proboscis (Figure 14)	***Nais pseudobtusa***
17a (15b)	Prostomium without proboscis	**18**
17b	Prostomium with elongate proboscis (Figure 15)	***Pristina leidyi***
18a (17a)	Green epidermal glands	**Aeolosoma cf. variegatum**
18b	Red epidermal glands (Figure 16)	**Aeolosoma cf. quarternarium**

**Figure 1.**
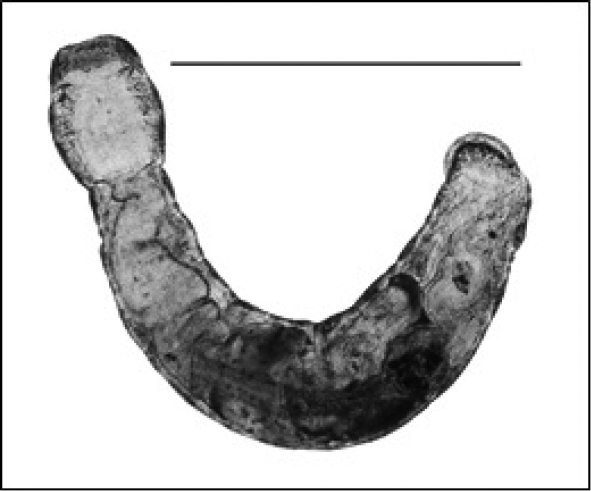
Branchiobdellida from crayfish host (Cambaridae) (scale 1 mm).

**Figure 2.**
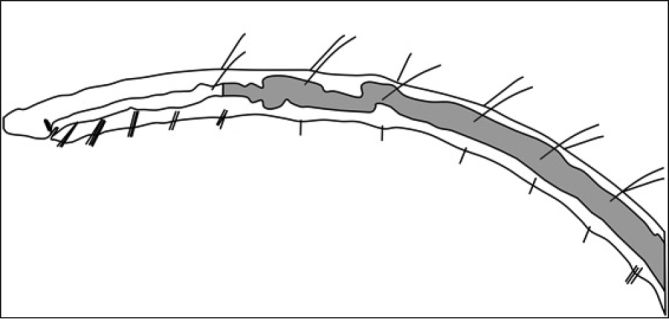
Drawing of generalized aquatic oligochaete showing anterior end and example positions of dorsal and ventral chaetae.

**Figure 3.**
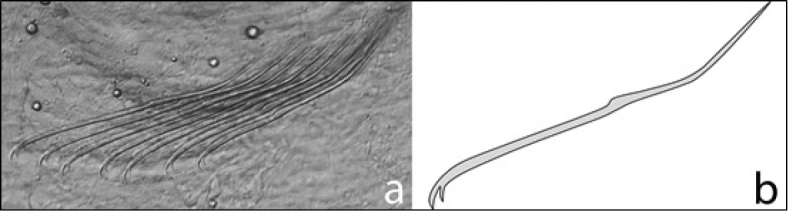
*Chaetogaster*: **A** photograph of typical bifid ventral chaetal bundle **B** drawing showing shape of one chaeta.

**Figure 4.**
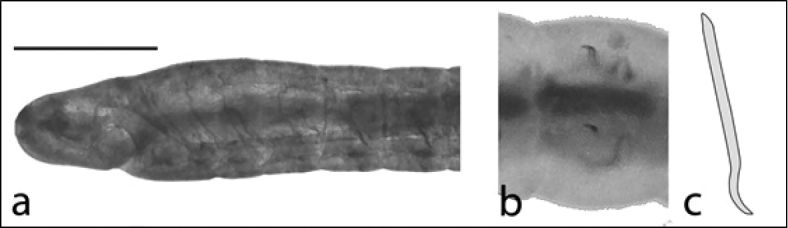
Haplotaxis cf. gordioides: **A** lateral view of anterior end showing prostomium and ventral mouth (scale 750 µm) **B** ventral view of one segment showing the two single ventral chaetae **C** drawing of one ventral chaeta.

**Figure 5.**
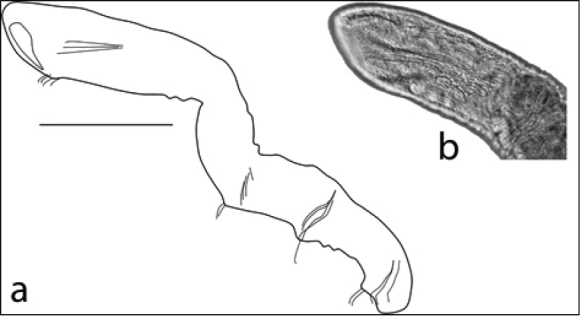
Chaetogaster cf. diaphanus: **A** drawing of entire body (scale 250 µm) **B** photo of anterior end showing prostomium protruding forward from mouth.

**Figure 6.**
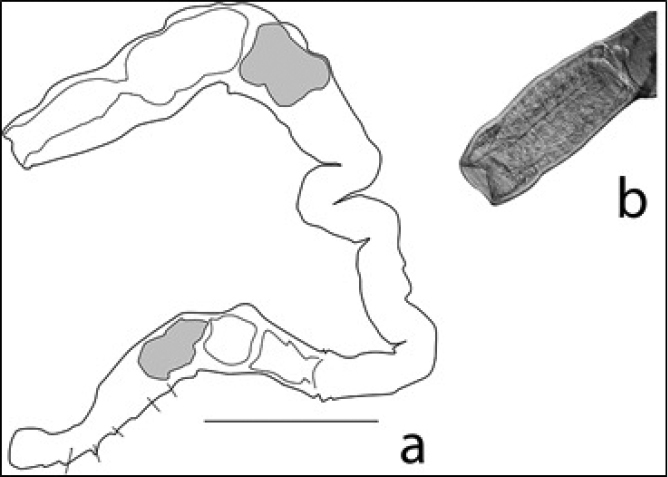
Chaetogaster cf. crystallinus: **A** outline drawing of entire body showing positions of chaetae that are limited to only ventral bundels of segments (scale 1 mm) **B** photo of anterior end showing cleft in prostomium.

**Figure 7.**
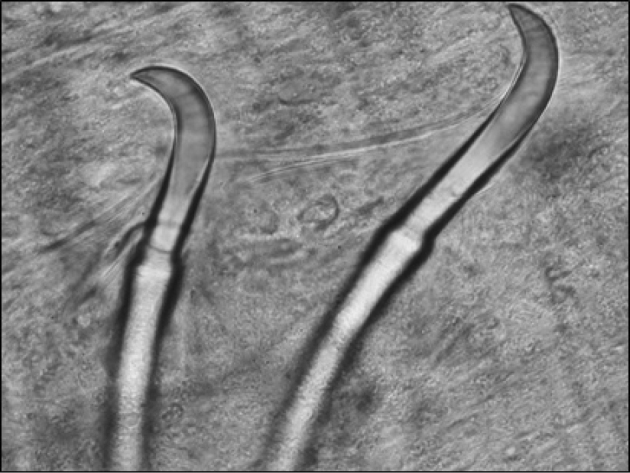
Paired chaetae typical of both dorsal and ventral bundles found on several lumbriculid taxa.

**Figure 8.**
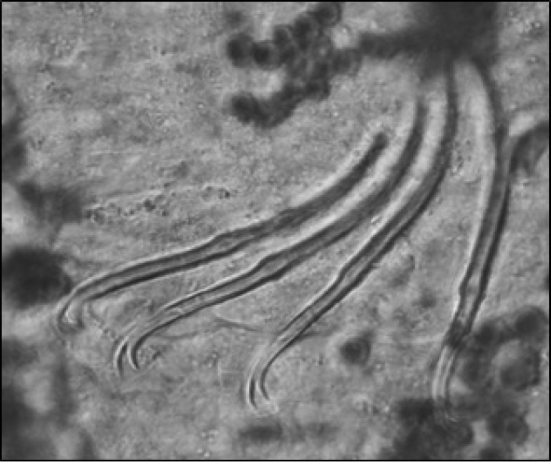
Multiple bifid ventral chaetae.

**Figure 9.**
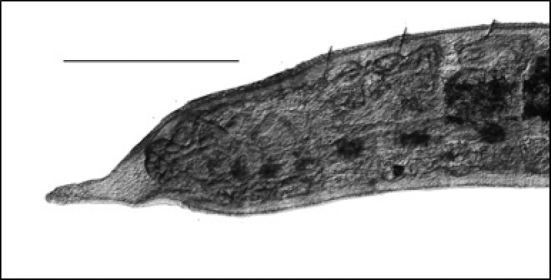
Lumbriculidae sp_1_: lateral photo of anterior end showing prostomium with conspicuous proboscis (scale 500 µm).

**Figure 10.**
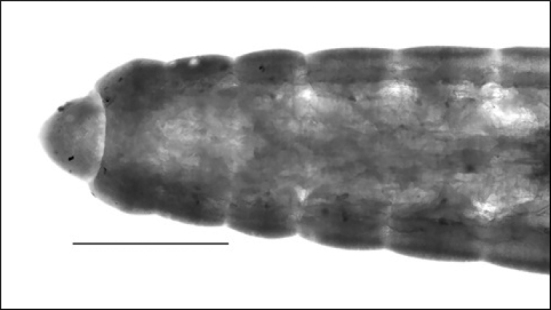
Lumbriculidae sp_2_: photo showing inconspicuous prostomium.

**Figure 11.**
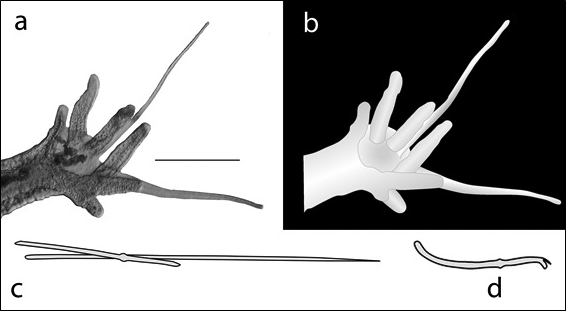
Dero (Aulophorus) furcatus: **A** photo of posterior end showing digitiform gills and elongate palps (scale 250 µm) **B** drawing of A **C** drawing of typical chaetae bundle **D** drawing of typical ventral chaeta.

**Figure 12.**
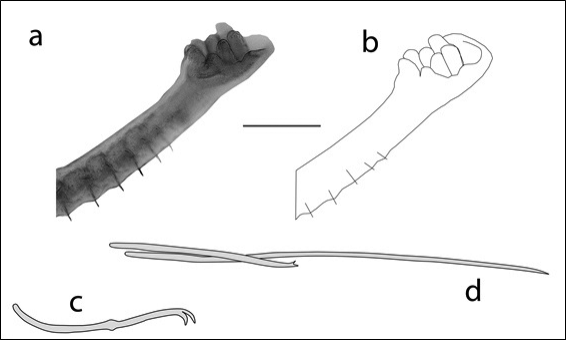
Dero (Dero) obtusa; anterior end and typical chaetae: **A** photo of posterior end showing gill fossa (scale 250 µm) **B** outline drawing of A **C** drawing of typical dorsal chaetae bundle **D** drawing of typical ventral chaeta.

**Figure 13.**
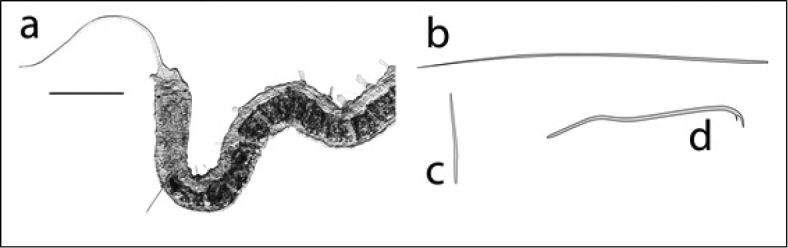
*Stylaria lacustris*, showing elongate prostomial proboscis, eyes, and typical chaetae: **A** photo of anterior end (scale 500 µm) **B** drawing of dorsal “hair” **C** drawing of dorsal “needle” **D** drawing of ventral chaeta.

**Figure 14.**
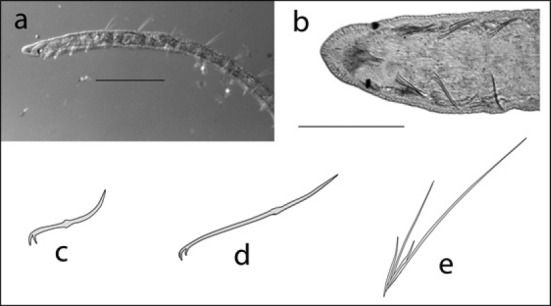
*Nais pseudobtusa*: **A** lateral photo of anterior end showing arrangement of chaetae, eyes, and overhanging prostomium (scale 500 µm) **B** dorsal photo of anterior end (scale 250 µm) **C** drawing of typical posterior-ventral chaeta **D** drawing of typical anterior-ventral chaeta **D** drawing of typical bundle of dorsal chaetae.

**Figure 15.**
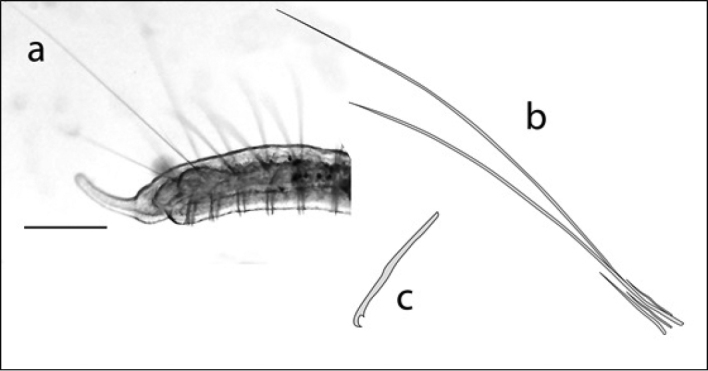
Pristina cf. leidyi: **A** lateral photo of anterior end showing elongate proboscis (scale 200 µm) **B** drawing of typical bundle of dorsal chaetae **C** drawing of typical ventral chaeta.

**Figure 16.**
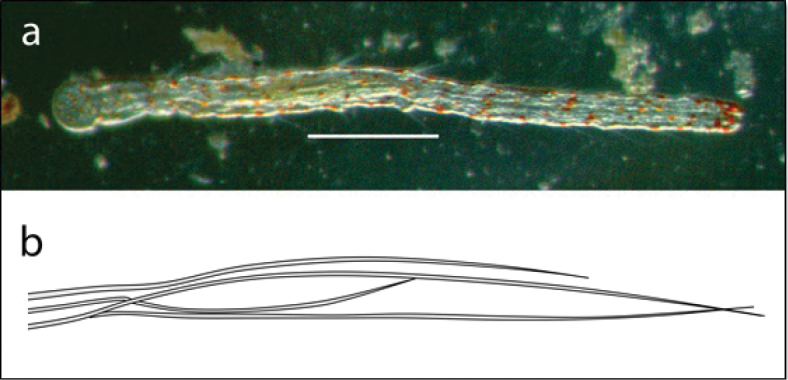
Aeolosoma cf. quarternarium: **A** photo of entire body showing red epidermal glands and disc-like anterior (scale 200 µm) **B** typical variable bundle of chaetae.

## Discussion

The annelids of the SMR headwaters, not surprisingly, proved to be quite diverse. The majority of this diversity was contained within the family Naididae. The naidid annelids that were identified belong to globally common and widely distributed genera and species (Brinkhurst and Jamieson 1971, Martin et al. 2008, Christoffersen 2010, Pinder 2010, Park and Yeon 2013). Though some showed slight morphological differences from published descriptions, these differences did not seem great enough to conclude that they might be new species.

Haplotaxis cf. gordioides (Family Haplotaxidae) was only collected from spring outflows, and the genus is known globally to be exclusively a groundwater taxon with cryptic microdiversity (Wetzel and Taylor 2001). This collection documents a new stygobiont from the region.

The Family Lumbriculidae may be even more specious in the SMR than indicated herein, as there were four distinguishable forms of lumbriculids collected during this study. However, it could not be determined whether or not the smaller two forms were juvenile forms of the larger two forms. Only the larger two forms are reported herein. Neither of these lumbriculid species could be confidently assigned to any known genus, and it is possible that they represent undescribed endemic species. Along with the collection of two species of Aphanoneura, the occurrence of the lumbriculids is highly suggestive that the SMR headsprings is an ancient habitat, as the members of both of these taxa are typically collected from ancient lakes (Martin 1996). One of the lumbriculids (referred to here as Lumbriculidae sp1) was also found to contain larvae of a trichosomoid nematode, as determined by the presence of a stichosome. Therefore, this lumbriculid species is thought to be serving as the intermediate host in the life cycle of a potentially undescribed trichosomoid.

Species of *Helobdella* leeches are typically found free living on the benthic sediments hunting for small arthropods, mollusks, and oligochaetes (Kutschera et al. 2013). Interestingly, a few specimens from this group were found attached to largemouth bass (*Micropterus salmoides* Lacépède). The method of attachment was quite bizarre. Individual leeches were connected to the ventral anterior surface of the bass with a single point of attachment, and the rest of the worm was enclosed in a mesh-like sack that dangled from the point of attachment. This finding represents an interesting note of life history for this group, as it seems they can also be facultative parasites; however, this is not the first report of *Helobdella* leeches parasitizing vertebrates (Platt et al. 1993, Tiberti and Gentilli 2010, Zimić 2015) but is the first report of this genus parasitizing fish that we are aware of.

Two additional oligochaete taxa were collected but have not been included herein because only one specimen of each taxon was collected and specimens were not in suitable condition for identification. A species of leech, which was only rarely collected from turtles, was also not identified. Neither of these oligochaetes or the leech were included in our results. Throughout specimen collections, numerous different forms of soil- and vegetation-dwelling nematodes were also collected. We did not attempt to identify any of these specimens. However, the variety of forms collected suggests that free-living nematodes may be the most specious group of soft-bodied metazoans in the SMR headwaters. The study of the SMR nematode fauna would represent a great contribution to what is known of the invertebrate fauna in this habitat.

Kånneby and Wicksten (2014) noted the collection of a new gastrotrich of the enigmatic genus *Redudasys* (Gastrotricha: Macrodasyida) from the SMR headsprings. Theirs is the first report of this genus from the Northern Hemisphere. We also collected gastrotrichs from the SMR, but they were identified to the genus *Chaetonotus* (Gastrotricha: Chaetonotida).

The identifications presented herein represent the first work on identifying annelids of the SMR and all of Central Texas. Therefore, we cannot speculate about how the diversity of the annelid fauna in the SMR compares to that of other Texas rivers. Greater taxonomic resolution could be achieved through genotyping specimens and we suspect that this would likely reveal appreciable cryptic diversity. Because this is the first annelid study in Central Texas we are hopeful that this will stimulate further research and lead to genotyping and further morphological studies by other authors in the SMR and other bodies of water. Even from the perspective of our incomplete survey, there seems to be compelling evidence that there is much more diversity in the SMR headwaters yet to be described, particularly for the invertebrate fauna.
